# Oxygen production via nitric oxide dismutation in diverse ammonia oxidizers

**DOI:** 10.1093/ismeco/ycag171

**Published:** 2026-06-22

**Authors:** Elisa Hernández-Magaña, Muzi Li, Alyson E Santoro, Donald E Canfield, Beate Kraft

**Affiliations:** Nordcee, Department of Biology, Faculty of Sciences, University of Southern Denmark, Odense, 5230, Denmark; Department of Ecology, Evolution and Marine Biology, University of California, Santa Barbara, CA, 93106, United States; Department of Ecology, Evolution and Marine Biology, University of California, Santa Barbara, CA, 93106, United States; Nordcee, Department of Biology, Faculty of Sciences, University of Southern Denmark, Odense, 5230, Denmark; Nordcee, Department of Biology, Faculty of Sciences, University of Southern Denmark, Odense, 5230, Denmark

**Keywords:** ammonia-oxidizing archaea, ammonia oxidizing bacteria, dark oxygen production, nitrous oxide, NO-dismutation, oxygen depletion

## Abstract

Ammonia-oxidizing archaea (AOA) are widespread and highly abundant in nature. Despite their typical aerobic metabolism, they thrive in ecosystems where oxygen is scarce. Recently, the AOA *Nitrosopumilus maritimus* SCM1 was shown to produce oxygen and N_2_O from nitrite upon oxygen depletion, with N_2_O subsequently converted to dinitrogen (N_2_) supporting nitric oxide (NO) dismutation as the proposed metabolic pathway. Here, we explore the ability of other ammonia oxidizers, with diverse phylogenetic affiliations and from different environmental settings to produce oxygen via NO-dismutation. We studied three marine AOA, one soil AOA, and two soil ammonia-oxidizing bacteria (AOB). Upon oxygen depletion, all strains accumulated oxygen. In incubations of the AOA strains with ^15^N tracers, two of them, *Nitrosopumilus piranensis* D3C and *Nitrosopumilus adriaticus* CCS1, showed transient ^46^N_2_O accumulation followed by linear ^30^N_2_ production, similar to SCM1, while *Nitrosopumilus adriaticus* NF5 and *Nitrososphaera viennensis* EN76 mainly produced ^46^N_2_O from nitrite without further N_2_ accumulation. These findings indicate that oxygen production through NO-dismutation is a common metabolism in cultured representatives of AOA, albeit with physiological variations between different strains such as the reduction of N_2_O to N_2_. Comparative genomics did not reveal a distinct putative N₂O reductase in the AOA strains with linear N_2_ production, suggesting that the observed physiological differences may not directly stem from gene inventory. The finding of oxygen production in several AOA, as well as AOB, suggests that dark oxygen production is a common trait among archaeal and bacterial nitrifiers and adds a potential explanation for their abundance in oxygen-depleted environments.

## Introduction

Ammonia-oxidizing archaea (AOA) and ammonia-oxidizing bacteria (AOB) perform the first step in the nitrification process in which they oxidize ammonia (NH_3_) to nitrite (NO_2_^−^) [1–3]. Ammonia oxidizers are widespread in a variety of environments such as soils, hot springs, wastewater treatment systems and freshwater and marine ecosystems including sediments and the water column [3–5]. Most AOB belong to the Betaproteobacteria and Gammaproteobacteria, while AOA belong to the phylum *Nitrososphaerota* (*Thaumarchaeota*) [4, 5], or, according to Genome Taxonomy Database, the phylum Thermoproteota, class Nitrososphaeria [6, 7].

Both AOA and AOB oxidize ammonia to hydroxylamine and further on to nitrite using oxygen. The first step is catalyzed by an ammonia monooxygenase (AMO) [8, 9]. AMO requires molecular oxygen for activation, which has been proven directly in AOB like *Nitrosomonas europaea* through stable isotope assays [10–12], and indirectly in AOA through stable isotope tracing of oxygen atoms from H_2_O into NO_2_^−^ [13, 14].

Due to the crucial role of oxygen in ammonia oxidation, a decrease in its concentration influences AOB physiology, and oxygen concentrations below 25 μmol·l^−1^ can negatively affect AOB growth [15]. In the case of the AOA *N. maritimus,* growth ceased under anoxia (see supplementary of reference [16]). Extensive research on the metabolism of AOB upon the onset of anoxia shows that they can switch to the anaerobic process of nitrifier denitrification (reviewed in reference [17]), producing NO, N_2_O [18] and in some cases N_2_ as end product [19–21]. In addition, while *N. maritimus* SCM1 and *Nitrosopumilus ureiphilus* PS0 sustain ammonia oxidation at oxygen concentrations down to around 10 μM [22], ammonia oxidation rates measured with standard colorimetric assays were not detectable below 1 μM of oxygen, [22, 23] (see supplementary information therein). However, AOA seem to be well adapted to oxygen-depleted environments such as oceanic oxygen minimum zones (OMZs) and sustain ammonia oxidation rates at nanomolar concentrations of oxygen [24].

Despite their requirement for oxygen, AOA are often abundant in oxygen-depleted or anoxic marine environments. In the Eastern Tropical South Pacific (ETSP) OMZ, archaeal *amoA* reached abundances comparable to oxic depths of about 2.7 x 10^6^copies/l [25]. In many cases, AOA peaked in oxygen-depleted waters respect to oxic shallower depths, for example in the Black Sea, where *Nitrosopumilus*-like AOA were up to tenfold more abundant in anoxic (2 × 10^8^ 16S rRNA gene copies/l) than in oxic depths [26]. Similarly, in parts of the Baltic Sea, AOA cell numbers were 15%–20% higher in oxygen-depleted compared to oxic depths [27], and in the Arabian Sea, ETSP [28] and the Eastern Tropical North Pacific [29] *amoA* genes have been found to be 2 to 10 times more abundant in the OMZ cores than in oxic depths. The abundance of AOA in the absence of detectable oxygen is enigmatic and sometimes explained as result of oxygen intrusions or local production of oxygen by phototrophs [30, 31].

The mechanisms by which AOA cope with oxygen depletion or anoxia remained unclear until the recent description of a previously overlooked pathway. In this pathway, upon oxygen depletion, nanomolar concentrations of oxygen (O_2_) and dinitrogen (N_2_) were produced by the AOA *Nitrosopumilus maritimus* SCM1 [32]. The proposed mechanism is a dismutation of nitric oxide (NO) to O_2_ and nitrous oxide (N_2_O), which is exergonic (ΔG0′ = −165 kJ·mol^−1^) [32].


(1) \begin{eqnarray*} 4N{O}_2^{-}+8{H}^{+}+4{e}^{-}\ \overset{NirK}{\longrightarrow}\ 4 NO+4{H}_2O \end{eqnarray*}

(2) \begin{eqnarray*} 4 NO\longrightarrow{O}_2+2{N}_2O \end{eqnarray*}

(3) \begin{eqnarray*} 2{N}_2O+4{H}^{+}+4{e}^{-}\longrightarrow 2{N}_2+2{H}_2O \end{eqnarray*}

In the proposed pathway of O_2_ and (ultimately) N_2_ production in *N. maritimus*, NO_2_^−^ is reduced to NO by the NirK nitrite reductase (Eq. 1). NO is dismutated into O_2_ and N_2_O (Eq. 2), which is further reduced to N_2_ (Eq. 3). Despite the physiological evidence for the pathway, the enzymes catalyzing the NO-dismutation and the N_2_O reduction steps have not yet been identified.

A NO dismutation pathway has been observed previously in the methane-oxidizing bacterium ‘*Candidatus* Methylomirabilis oxyfera’, where NO_2_^−^ is reduced to NO by a NirS nitrite reductase, and two NO molecules are subsequently dismutated into N_2_ and O_2_. In this pathway, the intracellularly produced O_2_ is used for methane oxidation [33–35]. In contrast to the archaeon *N. maritimus, Ca* M. oxyfera produces N_2_ directly in the dismutation step and does not accumulate N_2_O.

While the physiology of NO-dismutation has been studied in detail in *N. maritimus* [32, 36] it is still unknown how widespread this pathway is among nitrifiers. Furthermore, the enzymatic machinery for NO-dismutation remains unidentified, making the potential distribution of the pathway in the environment, and its ecological importance, difficult to explore through genomic analysis. Therefore, the objective of this study is to explore the oxygen production through NO-dismutation by different AOA strains, as well as in other nitrifiers not phylogenetically related to *Nitrosopumilus*. To this end, we assessed the physiology of four AOA and two AOB strains and their potential to produce oxygen under oxygen depletion. We used trace-range oxygen sensors (nanomolar range) and incubations with ^15^N labelled substrates. We also compared the genomes of the AOA strains we explored, in search for genes that could potentially explain the physiological differences observed.

## Materials and methods

Oxygen production in the dark coupled to nitrous oxide and dinitrogen production was tested under oxygen-depletion in incubations of four marine AOA strains: *Nitrosopumilus piranensis* D3C, *Nitrosopumilus adriaticus* NF5, [37, 38]*, Nitrosopumilus adriaticus* CCS1 [39], and *N. maritimus* SCM1 [40], previously tested in [32]. The soil AOA *Nitrososphaera viennensis* EN76 [41] and the soil AOB strains *N. europaea* Nm50 and *Nitrosospira multiformis* Nl13 were tested as well*.*

The marine AOA strains were grown in HEPES buffered (pH 7.6) Synthetic Crenarchaeota Medium, at their respective optimal temperature (Table 1), with the addition of catalase (10 units/ml) [42]. *Nitrososphaera viennensis* EN76 was maintained in freshwater medium (FWM) for soil archaea, with the addition of pyruvate (1 mM) [43, 44]. The AOB strains were grown in HEPES-buffered (pH 7.6) basal mineral salt medium [45] at 28°C. All were grown in axenic batch cultures in the dark without stirring (details in supplementary).

**Table 1:** Strains utilized for batch experiments and culture conditions.

Strain	Temperature (°C)	Culture media	Reference
*Nitrosopumilus piranensis* D3C	30	SCM HEPES buffered (pH 7.6)	[37, 38]
*Nitrosopumilus adriaticus* NF5	30	SCM HEPES buffered (pH 7.6)	[37, 38]
*Nitrosopumilus adriaticus* CCS1	25	SCM HEPES buffered (pH 7.6)	[39]
*Nitrososphaera viennensis* EN76	42	FWM for soil AOA JMC	[43]
*Nitrosomonas europaea*	28	Medium 1583, DSMZ	[45]
*Nitrosospira multiformis*	28	Medium 1583, DSMZ	[45]

Batch cultures that had oxidized at least 95% of the initial NH_4_^+^ (1 mM) were used for incubations under oxygen depletion. Prior to the incubations, all cultures were grown aerobically. Each batch culture was sparged with argon gas (99.999% purity) for 45 min to reduce the oxygen concentration and aseptically transferred by overpressure into custom-made 330 mL glass bottles [46] as described previously in [36] and detailed in the supplementary material.

Bottles were constantly stirred with glass-coated stirring bars (VWR, UK) at 300 rpm submerged in a water bath at the respective growth temperature of each strain for 72 h (Table 1). Oxygen was monitored constantly during the incubation with trace fluorescence oxygen sensors (hereafter optodes) with a lower detection limit of 0.5 nmol·l^−1^ [47]. NO concentrations were continuously monitored with NO microsensors (Unisense, Denmark) and all oxygen concentration measurements were corrected for NO interference following [32].

AOA batch cultures with a pool of ^15^N labelled NO_2_^−^ (1 mM) were used to evaluate ^30^N_2_ and ^46^N_2_O production, the product and intermediate of the proposed archaeal NO dismutation pathway (Eq. 1–3) [32]. The ^15^NO_2_^−^ pool was generated by growing the batch culture that was later used for the incubations with ^15^N-labelled NH_4_^+^. Once more than 95% of the ^15^NH_4_^+^ was completely oxidized to ^15^NO_2_^−^ during aerobic growth, the culture was used for oxygen-depleted incubations. Immediately before starting the incubations, 500 μM of ^14^NH_4_^+^was added to the batch culture to ensure sufficient ammonia for ammonia oxidation during the experiment and to trap any residual amounts of ^15^N-NH_4_^+^ that could have been left. Therefore, the only relevant source of ^15^N in the incubation was ^15^N-NO_2_^−^. An exception to this were the *N. piranensis* D3C and *N. viennensis* batch culture incubations, which due to the preculture growth conditions, had an initial pool of 1 mM ^15^NO_2_^−^, and 200 μM ad 50 μM ^14^NO_2_^−^, respectively. As the other cultures, 500 μM ^14^NH_4_^+^ was added prior to the start of the incubation (more details in the supplementary material).

The AOB strains tested are known to produce N_2_O [18] and/or N_2_ from nitrite upon oxygen-depletion [20, 21] so far attributed to nitrifier denitrification. Therefore, AOB strains were not tested for ^46^N_2_O production from ^15^NO_2_^−^ during oxygen-depletion.

Controls for abiotic reactions with dead cells and the AOA culture media used here have been reported previously for a *Nitrosopumilus* strain in the same experimental set up, [32]. In the present study, dead-cell controls of *N. viennensis* growing in FWM were produced by adding 3 ml of saturated HgCl_2_ solution in three incubation bottles after oxygen was depleted (Fig. S3B) to discard abiotic production of oxygen.

Samples were collected at 0, 8, 24, 32, 48, 56, and 72 h with gas-tight syringes (Hamilton, USA) connected to stainless steel needles (Ochs, Germany) through the capillary of the incubation bottles. To avoid headspace formation in the incubation bottles, the volume collected was simultaneously replaced with sterile deoxygenated culture media (sparged with argon). Despite the precautions, a slight intrusion of oxygen when collecting samples could not be avoided.

The samples were collected without a headspace in gas-tight 3 ml exetainers and preserved with 50 μl of saturated HgCl_2_ solution. N_2_ and N_2_O were analysed by coupled gas chromatography–isotope ratio mass spectrometry (GC-IRMS) on a Thermo Delta V Plus isotope ratio mass spectrometer [48].

We used comparative genomics to search for the genes potentially responsible for dinitrogen production. OrthoFinder (v2.5.5) [49] was applied to identify shared and unique orthologs across the five AOA strains. Cupredoxin domain-containing proteins were detected by OmicsBox (v3.0.30) [50] built-in InterProScan [51] with default settings. Subsequently, MAFFT (v7.520) [52] and FastTree using JTT + CAT model and default settings (v2.1.11) [53] were utilized to align the cupredoxin proteins and construct the phylogenetic tree, respectively (Fig. S6).

## Results

### Oxygen production in AOA

**Figure 1:**
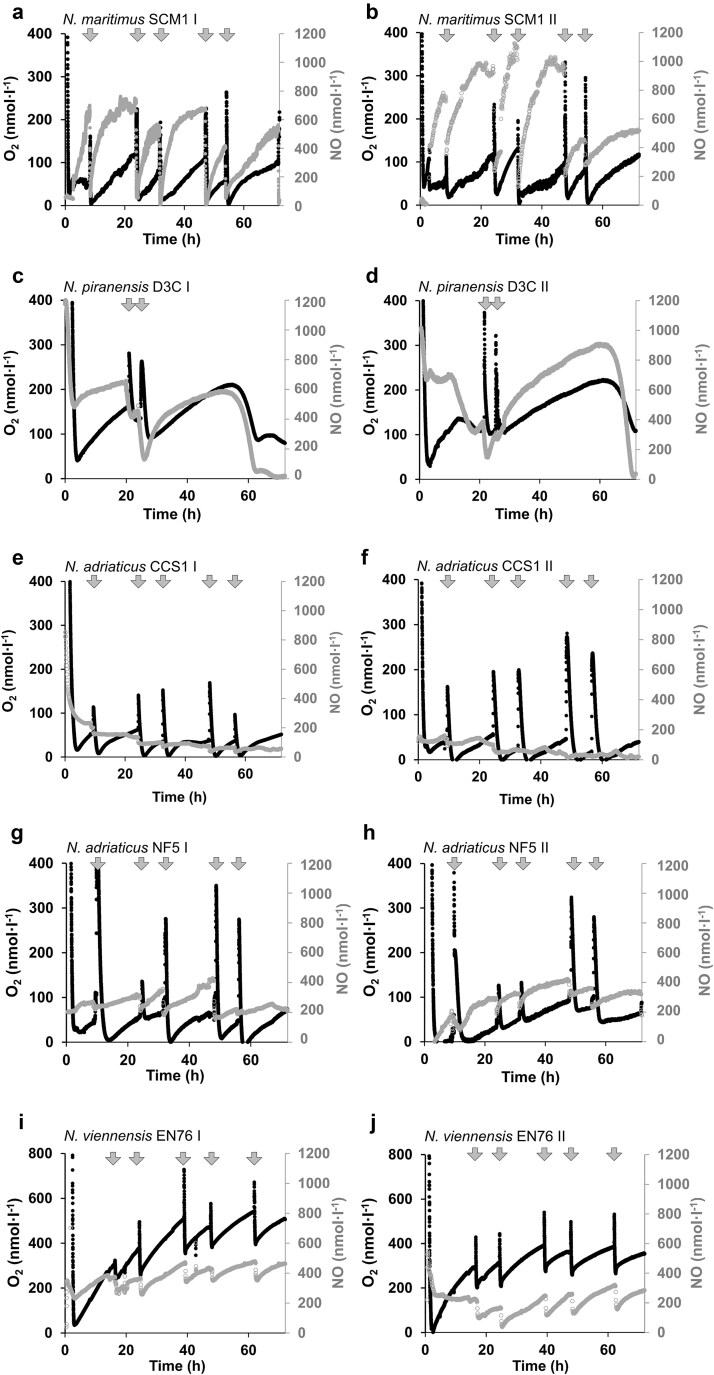
Oxygen accumulation coupled to NO accumulation in oxygen-depleted incubations of AOA strains. Oxygen concentrations are corrected for interference with NO. (a, b) *Nitrosopumilus maritimus* SCM1 for which oxygen production was reported previously in [32]. (c, d) *Nitrosopumilus piranensis* D3C. (e, f) *Nitrosopumilus adriaticus* NF5. (g, h) *Nitrosopumilus adriaticus* CCS1. (i, j) *Nitrososphaera viennensis* EN76. The figure shows two reproducible replicates of each strain (at least *n* = 3 per incubation), the rest of the replicates are shown in supplementary Figs S2 and S3. Pronounced increases in the oxygen concentration (marked with arrows) are due to oxygen intrusion by sample collection (see main text).

We performed incubations under oxygen depletion for three marine AOA strains from the clade *Nitrosopumilales* (Group I.1a) and one soil AOA strain from the clade *Nitrososphaerales* (Group I.1b) (Fig. 1). Oxygen consumption was immediately observed once the incubation started, indicative of active oxygen respiration at nanomolar oxygen concentrations (Table 2) and confirming that the activity of the batch cultures was not affected by purging with argon or by the reduced oxygen concentrations (Fig. 1, first 5 h of incubation).

**Table 2:** Oxygen net accumulation and respiration rates for the different AOA and AOB strains.

Strain	O_2_ accumulation rate (nmol·l^−1^·h^−1^)^a^	Cell specific O_2_ accumulation rate (amol·cell^−1^·h^−1^)	O_2_ respiration rate (nmol·l^−1^·h^−1^)	Cell specific O_2_ respiration rate (amol·cell^−1^·h^−1^)	Cells densities (cells·ml^−1^)	Volume (μm^3^)	Surface area/volume ratio (SA/V)
*Nitrosopumilus maritimus* SCM1	13.27 ± 8	0.20 ± 0.12	409.47 ± 122.83	6.02 ± 1.81	6.8 × 10^7^ ± 4.5 × 10^6^	0.01	23.37 ± 2.92
*Nitrosopumilus piranensis* D3C	7.44 ± 2.22	0.21 ± 0.06	256.88 ± 17.61	7.16 ± 0.49	3.59 × 10^7^ ± 9.3 × 10^6^	0.03	20.53 ± 2.83
*Nitrosopumilus adriaticus* NF5	3.99 ± 0.90	0.09 ± 0.02	446.92 ± 107.52	9.6 ± 2.326	4.63 × 10^7^ ± 1.5 × 10^7^	0.04	19.54 ± 2.57
*Nitrosopumilus adriaticus* CCS1	7.13 ± 0.75	0.19 ± 0.02	349.07 ± 64.47	13.59 ± 2.51	2.57 × 10^7^ ± 6.4 × 10^6^	NA	NA
*Nitrososphaera viennensis* EN76	21.12 ± 1.77	0.70 ± 0.06	2285.21 ± 376.98	75.46 ± 12.45	3.03 × 10^7^ ± 3 × 10^6^	0.07	9.23 ± 1.85
*Nitrosomonas europaea*	18.51 ± 6.58	5.25 ± 1.94	612.99 ± 427.45	173.79 ± 121.19	3.53 ×10^6^ ± 1.2 × 10^6^	0.50	5.69 ± 0.9
*Nitrosospira multiformis*	8.23 ± 4.76	2.44 ± 1.41	186.67 ± 88.70	55.22 ± 26.24	3.38 × 10^6^ ± 3.2 × 10^5^	0.75	5.44 ± 0.63^b^

Oxygen net accumulation rates were calculated for the first 3 hours after oxygen accumulation started. Oxygen respiration rates were calculated for 30–60 min prior to the minimum oxygen concentration reached before oxygen accumulation.

Values (except for volume) are shown as average (at least *n* = 3) ± standard deviation.

^a^ Oxygen accumulation maximum values mentioned in the text were taken from around 12 h after the oxygen production started unless otherwise stated.

^b^ SA/V from *Nitrosospira briensis*, included here as reference. The volume was calculated from the length and diameter reported in Table S4 of [65]. NA: Not Available.

After oxygen consumption, oxygen accumulation was observed in all the AOA strains tested and was coupled to NO-accumulation (Fig. 1). Abrupt increases in oxygen concentration during incubations, hereafter referred to as oxygen pulses, were a consequence of sample collection or injection of oxygenated water (see Materials and methods, marked with arrows in Fig. 1). This oxygen was immediately consumed, after which oxygen accumulation started again.

We note that the accumulated oxygen (net production) is the result of the net balance between the total oxygen produced (gross production) and simultaneous oxygen consumption by cell respiration (including ammonia oxidation). Oxygen accumulation rates of about 7 nmol·l^−1^·h^−1^ were reached for the two marine strains *N. piranensis* D3C and *N. adriaticus* CCS1, while *N. adriaticus* NF5 showed a slightly lower accumulation rate (4 nmol·l^−1^·h^−1^, Table 2). The soil *N. viennensis* EN76 accumulated oxygen at the highest rate among all the examined AOA strains (21 nmol·l^−1^·h^−1^, Table 2). *N. viennensis* EN76 also showed the highest respiration rate prior to oxygen accumulation.

The oxygen accumulation rates normalized by cell numbers followed a similar pattern, as the cell densities in all the AOA batch cultures were within the same order of magnitude during the incubation [between 3 to 7 × 10^7^ cells·ml^−1^ (Table 2)].

Oxygen accumulated to different levels among the AOA strains, within a range of 50 to 600 nmol·l^−1^. The marine strain *N. adriaticus* CCS1 was at the low end of the range, while the soil strain *N. viennensis* was at the high end. Controls with dead cells did not show any evidence of abiotic oxygen production (Fig. S3B) [32].

The minimum oxygen concentration at which oxygen accumulation began varied among strains, and in some cases, among incubation replicates. In most cases, oxygen was drawn down to concentrations close to the optodes’ detection limit (0.5 nmol·l^−1^) [47], followed by oxygen accumulation, similar to what was observed for *N. maritimus* (Fig. 1a,b) [32]. However, *N. piranensis* and *N. viennensis* showed marked differences to this pattern.

In incubations of *N. piranensis*, the lowest oxygen concentrations reached by oxygen respiration, before oxygen accumulation, were always above 38 nmol·l^−1^. In the incubations of *N. viennensis*, the lowest oxygen concentration was reached prior to the first oxygen accumulation and within the first 5 h of the incubation. From the second sampling point onwards, and after oxygen pulses during sampling and associated oxygen drawdown, oxygen was only consumed to concentrations of between 200 and 390 nmol·l^−1^ before oxygen accumulated again (Fig. 1i,j).

In general, the amount of oxygen introduced in the incubation bottles during sample collection had an influence on the minimum concentration reached before the oxygen accumulation started again, suggesting that a higher oxygen pulse triggered a more pronounced net oxygen consumption. For example, in the *N. adriaticus* NF5 incubations (Fig. 1g and h), smaller oxygen pulses were followed by less pronounced oxygen consumption, in contrast to the higher oxygen pulses that were followed by a more pronounced oxygen consumption. While the pattern was the same, the amount of oxygen that triggered complete consumption of oxygen varied among strains.

The effect of different oxygen pulse sizes was explored in more detail in incubations with *N. maritimus* SCM1 (Fig. S1). The injection of different volumes of sterile oxygenated medium to incubation bottles also showed that higher amounts of oxygen injected were followed by more pronounced oxygen consumption, thus supporting the observations described above.

Accumulation of nitric oxide was coupled to O_2_ accumulation in all AOA studied (Fig. 1), but with differences in the trends. In the marine AOA strains *N. maritimus, N. piranensis*, and *N. adriaticus* NF5*,* as well as in the soil AOA *N. viennensis,* NO accumulation was parallel to oxygen production. In the case of *N. adriaticus* NF5 and *N. viennensis*, NO accumulated to a maximum of 600 nmol·l^−1^, while in *N. maritimus* and *N. piranensis* NO concentrations exceeded 600 nmol·l^−1^ and sometimes reached 1000 nmol·l^−1^ or more. The lowest NO concentration accumulated among the strains was observed in the incubations with *N. adriaticus* CCS1 (up to 200 nmol·l^−1^). For this strain, NO concentrations declined three to four hours after the oxygen accumulation started, while oxygen accumulation continued.

### Oxygen production in AOB

**Figure 2:**
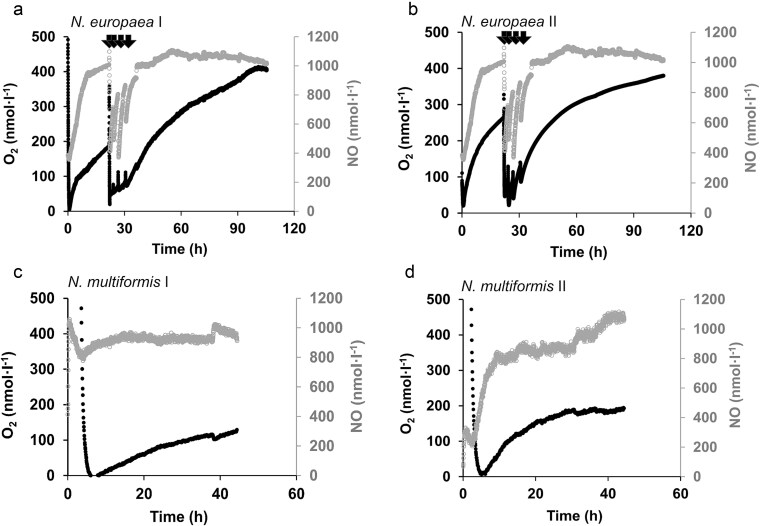
Oxygen accumulation coupled to NO accumulation in oxygen-depleted incubations of different AOB strains. (a, b) *Nitrosomonas europaea* (c, d) *Nitrosospira multiformis*. Two reproducible replicates of at least *n* = 3 per incubation are shown (other replicates shown in Fig. S4). In the case of incubations with AOB pronounced increases in the oxygen concentration (such as in a and b) are due to oxygen intrusion associated to fully oxygenated water injection in the incubation (black arrows). Oxygen concentrations were corrected for the interference of NO with the optodes.

Two AOB strains were incubated under oxygen depletion. Both, *N. europaea* and *N. multiformis* began consuming oxygen at the beginning of the incubation and drew the oxygen down to the detection limit of the optodes (Fig. 2). Similar to the AOA, after the oxygen was respired, oxygen accumulation was observed. The oxygen accumulation rate of *N. europaea* was twice as high as for *N. multiformis* (Table 2).

When the incubation remained undisturbed (without oxygen pulses), oxygen in the *N. europaea* incubation accumulated to above 300 nmol·l^−1^ (Fig. 2a and b), which was greater than for all the other strains tested (AOA and AOB). In the case of *N. multiformis* incubations, oxygen accumulated up to 100 or 200 nmol·l^−1^. Like the AOA incubations, after a sample was collected or an oxygen pulse was introduced, oxygen was consumed, and oxygen accumulation started again.

Cell densities in AOB cultures were lower than in AOA cultures (around 3 × 10^6^ AOB cells per ml, Table 2). Therefore, the oxygen accumulation rates per cell were around 10 to 20 times higher in the AOB cultures compared to the AOA. Similarly, the oxygen respiration rates per cell before oxygen accumulation were notably higher in the AOB strains than in the AOA strains (Table 2).

Just as in the AOA, NO accumulation was coupled to oxygen accumulation in the AOB strains tested (Fig. 2). NO concentration in incubations of *N. europaea* increased markedly when oxygen was completely consumed at the beginning of the incubation. After an oxygen pulse, NO concentration decreased, comparable to the observations in the AOA incubations. For both AOB strains, NO reached concentrations around 1000 nmol·l^−1^ and then remained relatively stable.

### N_2_ accumulation and N_2_O dynamics under oxygen depletion

**Figure 3:**
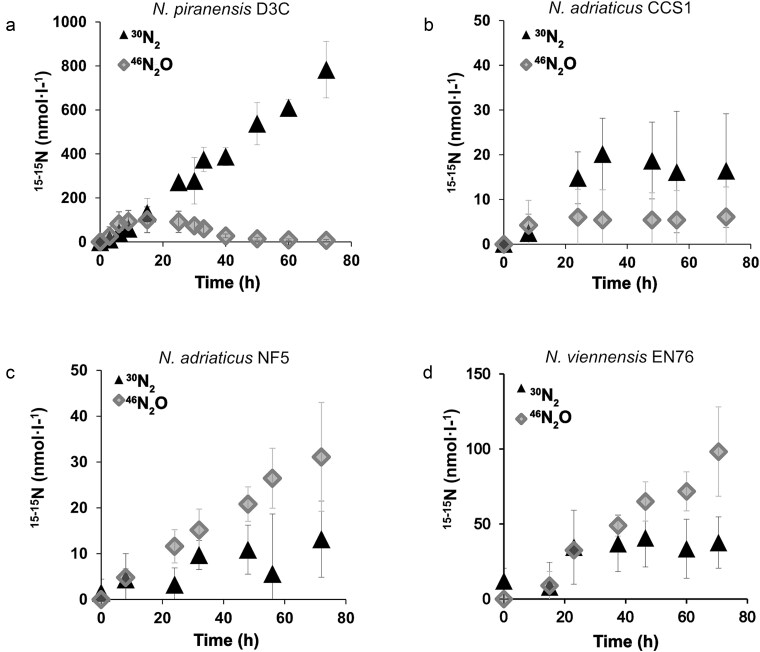
Nitrogen and nitrous oxide accumulation from nitrite during oxygen production by different AOA strains. ^30^N_2_ production (black triangles) and ^46^N_2_O accumulation (grey diamonds) from ^15^NO_2_^−^. Datapoints are an average of three replicates and error bars represent the standard deviation. Some error bars are smaller than the symbols and therefore not visible.

N_2_O and N_2_ production from NO_2_^−^ was tested in oxygen-depleted incubations amended with ^15^NO_2_^−^ in the marine AOA strains and in the soil AOA (Fig. 3). In these experiments, ^46^N_2_O accumulation (as an intermediate which then is further reduced to N_2_) and ^30^N_2_ production from ^15^NO_2_^−^ is expected if the AOA strains perform NO dismutation as proposed for *N. maritimus* [32]. Production of N_2_O from NO_2_^−^ by AOB such as *N. europaea* and *N. multiformis* during anoxia, has been reported before [18, 54] and attributed to nitrifier denitrification.

In the case of the marine AOA *N. piranensis* D3C and *N. adriaticus* CCS1*,* a transient accumulation (e.g. production followed by consumption) of ^46^N_2_O was observed in incubations amended with a pool of ^15^NO_2_^−^, and ^30^N_2_ accumulated linearly from the beginning of the incubation suggesting that ^15^NO_2_^−^ is transformed to ^46^N_2_O and then further into ^30^N_2_ (Fig. 3a and b), similar to the observations reported for *N. maritimus* [32, 36].

In contrast to the other marine AOA, *N. adriaticus* NF5 and the soil strain *N. viennensis* EN76 showed a linear increase of ^46^N_2_O from the beginning of the incubation while the simultaneous production of ^30^N_2_ was minor (Fig. 3c and d). To highlight the physiological differences, we will refer to these two strains as N_2_O producers and to strains with linear N_2_ production as N_2_ producers.

The production of ^46^N_2_O and ^30^N_2_ indicates that both nitrogen atoms come from nitrite and none from ammonium. Accumulation of ^29^N_2_ was only observed for the strain *N. piranensis*, for which the incubation was started with a mix of ca. 1 mM ^15^NO_2_^−^ and 200 μM ^14^NO_2_^−^ (Fig. S5A; see supplementary text). A slight increase of ^45^N_2_O was observed in the incubation of *N. viennensis* (Fig. S5D).

### Comparative genomics of N_2_ and N_2_O producing AOA strains

We compared the genomes of the five AOA strains tested to determine any differences in gene content that might explain the differences in strain physiology with respect to N_2_O and N_2_ production and consumption. In particular, we looked for the presence of putative nitrous oxide reductases unique to the N_2_-producing strains. We identified five orthogroups that were shared among the N_2_-producing strains (CCS1, D3C, and SCM1) but absent in the non-N_2_ producing strains (Table S1). However, none of these five orthogroups contains a cupredoxin domain (Table S2), present in known nitrous oxide reductases. Seven orthogroups are present exclusively in strains that did not produce N_2_ (Table S3), but again, no obvious connections between these orthologs and nitrogen redox cycling were apparent.

Nitrous oxide reductase is usually composed of two domains, CuA and CuZ (containing the catalytic centre). Each of the five AOA strains has only one cupredoxin-containing gene for the CuA centre of nitrous oxide reductase (IPR002429; CCS1: 2932131032, D3C: 2628582943, EN76: 2585919028, NF5: 2630188923, SCM1: 641315800) (Fig. S6). Thus, though CCS1 and NF5 have distinct capabilities of reducing N_2_O to N_2_, the sequences of their CuA-containing proteins are the same. CuZ domain-containing proteins were further examined using the InterProScan and OrthoFinder results. CuZ domain-containing proteins were only found in CCS1, NF5, and EN76 (IPR011045; CCS: 2932132464, NF5: 2630190368, EN76: 2585917421). The CuZ domains of the CCS1 and NF5 CuZ-containing proteins are identical. However, the CCS1 CuZ-containing protein has three cupredoxin domains at its N-terminal, while NF5 CuZ-containing protein has only two (Fig. S7), which may make the two proteins function differently. Moreover, the localization of the three CuZ-containing proteins (Fig. S8) differs among the strains; the NF5 CuZ-containing protein lacks a signal peptide, which possibly causes the protein (and its product) to remain inside the cell. EN76 CuZ-containing protein does not have cupredoxin domains (Fig. S7). Overall, no differences in canonical N_2_O reductase inventory between N_2_O and N_2_ accumulating strains were identified.

## Discussion

Oxygen production was observed in all the AOA strains tested, with representatives of the clades *Nitrosopumilales* from marine environments, and *Nitrososphaerales* from soils. The coupling of oxygen production to NO accumulation and the transient accumulation of N_2_O followed by N_2_ production (or in some cases only N_2_O production) indicate that oxygen production in these AOA strains likely proceeds via the same pathway of NO dismutation as has been proposed for *N. maritimus* SCM1 [32]. Overall, oxygen production is common in cultured representatives of AOA including all three tested *Nitrosopumilus* species, the dominant genus of AOA in marine oxygen-depleted waters [26, 55], and therefore has important implications for our understanding of the physiology of AOA under oxygen-depletion and may warrant reconsideration of their metabolic role in oxygen-depleted ecosystems. However, whether this capacity is expressed under environmental conditions remains to be established.

We observed interesting differences among strains regarding accumulation patterns and rates of oxygen and the nitrogen compounds. For example, *N. adriaticus* NF5 and *N. viennensis* EN76 produced N_2_O as the main end product with minor N_2_ production (Fig. 3c and g), in contrast to the other AOA, SCM1, D3C, and CCS1, which showed a transient N_2_O accumulation together with N_2_ production. These observations support NO-dismutation as the oxygen producing core step being conserved in all AOA tested: the dismutation of NO into N_2_O and O_2_ [32].

While our results indicate that the core steps of the pathway are the same in the different AOA strains, oxygen accumulation rates (net production) varied among them. The soil AOA *N. viennensis* EN76 showed a remarkably higher oxygen accumulation rate in comparison to the marine strains. As previously demonstrated, the oxygen produced by pure cultures of AOA sustains their main metabolism (ammonia oxidation) [32]. Therefore, the differences in oxygen accumulation rates among the tested strains indicate possible differences in the specific capability of the strains to both produce oxygen and to utilize it simultaneously.

The difference among the marine AOA in the minimum oxygen concentration reached after oxygen pulses and prior to oxygen production could point towards differences in the oxygen concentration threshold or range that triggers the switch in metabolism and onset of net oxygen accumulation. Facultative anaerobes possess oxygen-sensing transcriptional factors such FNR in bacteria or NarO in haloarchaea which control the transition from aerobic to anaerobic metabolism [56, 57]. No such oxygen-sensing factor has been identified in AOA so far, but the observed differences among strains may reflect different sensitivities of a possible regulator to changes in oxygen and/or nitric oxide.

The first step of the NO-dismutation pathway, the reduction of NO_2_^−^ to NO, is most likely performed by the nitrite reductase NirK, which is present in all the AOA strains tested [58, 59]. The enzymes involved in the NO dismutation and N_2_O reduction steps of the proposed pathway are still unknown and no NO-dismutases or complete putative N_2_O reductases have been identified so far in the available AOA genomes [59, 60].

While the N_2_O and oxygen accumulation are consistent among AOA strains, the lack of linear N_2_ production in some of them suggests that an enzyme responsible for N_2_O reduction is absent or inactive in these AOA. Comparison of the *N. adriaticus* strains NF5 and CCS1 is particularly useful in this regard, as the two strains share an average nucleotide identity of 97.94% [61] but differ in their apparent N_2_O consuming ability. While we found interesting differences in the structure and putative localization of the CuZ-containing proteins in these two strains, most probably they are not part of the N_2_O reductases involved in the metabolism of AOA under oxygen depletion as these domains are only present in CCS1, NF5, and EN76. Additional comparative genomic analysis of subtle differences in other cupredoxin-containing proteins may yield further insights. Furthermore, differences in N_2_O reduction capability among AOAs could be due not only to genomic differences but also to novel enzymes yet to be identified and annotated, structural differences, or differential expression.

AOA have been suggested to be important N_2_O producers in the open ocean, mainly in oxygenated waters, and therefore N_2_O production by AOA has mainly been attributed to their aerobic metabolism [13, 62, 63]. Under low oxygen concentrations, AOA contribute to N_2_O production as byproduct of ammonia oxidation [22, 64]. This study provides evidence that underlines the importance of AOA as potential N_2_O producers in oxygen-depleted environments, from NO_2_^−^ via NO-dismutation. Most importantly, the differences in N_2_O reduction capability between the AOA strains tested in the present study raises the question whether certain AOA groups in oxygen-depleted ecosystems constitute sources of N_2_O to the environment, or in some cases, potential sinks [36].

A major finding of this study is the oxygen accumulation by AOB. Though both AOB and AOA perform ammonia oxidation, they often occupy different niches mainly due to their different affinities for ammonium [16, 65]. Still, members of both groups can survive in oxygen-limited environments [25–27, 66–69]. AOB cope with oxygen-limitation by performing nitrifier denitrification, with some variations among species, such as differences in N_2_O accumulation [18, 68, 70] and N_2_ as final product [19–21]. Strikingly, we observed oxygen production by two phylogenetically distinct AOB strains in pure culture, *N. europaea* and *N. multiformis,* when oxygen concentrations reached the low nanomolar range. Thus, oxygen production under oxygen depletion occurs among unrelated bacterial ammonia oxidizers as well.

These results were unexpected, as despite more than 30 years of research on AOB under oxygen limitation and exposure to anoxia, no oxygen production has been reported in AOB to the best of our knowledge. On the other hand, the novelty of our results is based on the use of trace range oxygen sensors. The oxygen accumulation observed in the incubations of the present study reached a maximum of 100 to 300 nmol·l^−1^, while the oxygen sensors previously used in physiological studies had a detection limit in the range of 1 μmol·l^−1^ [19, 71]. In other studies, oxygen concentrations were not explicitly measured, not mentioned [20, 72], or only measured at the beginning and/or the end of the incubation [70, 73], leaving the possibility for changes in oxygen concentration during the incubation to be overlooked.

During oxygen depletion, production of ^46^N_2_O from ^15^NO_2_^−^ by the AOB *N. europaea* has been reported [18, 21] and identified as nitrifier denitrification. Oxygen accumulation by AOB coupled to NO and previously published evidence of N_2_O and N_2_ production from NO_2_^−^, suggests the possibility of a similar oxygen production pathway as in AOA. The isotopic signature of the nitrogen compounds produced during nitrifier denitrification is not distinguishable from those produced by NO-dismutation. Therefore, N_2_O production via NO-dismutation could easily have been overlooked and mistaken as nitrifier denitrification. Further research into the mechanism and genetics of oxygen production by AOB upon oxygen depletion is needed to confirm that the pathway in AOB is the same as in AOA.

There are multiple metabolic reactions that can generate nitrogenous gases in AOB. For example, *N. europaea* has the capability of producing NO through two mechanisms: aerobic hydroxylamine oxidation to NO by the hydroxylamine oxidoreductase and the reduction of NO_2_^−^ to NO by the NirK nitrite reductase. The latter is predominant at low-oxygen concentrations [74]. Most of the AOB available in culture have NOR and NirK. However, *N. europaea* NirK-deficient mutants can still perform nitrifier denitrification [21]. Evidence points towards a yet unknown nitrite reductase and holds questions about the function of NirK in the metabolism of AOB [17], especially under oxygen depletion.

No potential NO-dismutase or N_2_O reductase have been identified in AOB, even though N_2_ has been reported as product of the nitrifier denitrification pathway (reviewed in [75]). Considering the phylogenetic distance between AOB and AOA and the concomitant differences in their metabolic machineries, we cannot exclude that the enzyme(s) responsible for oxygen production in AOB may be different from the one(s) in AOA. Additionally, oxygen production has been proposed to occur in *Pseudomonas aeruginosa* [76], but there is not isotopic evidence supporting that the mechanism behind is the same as reported here for AOA and AOB.

AOA abundance and activity in oxygen-depleted ecosystems has been enigmatic and difficult to explain. Different explanations have been proposed for AOA abundance in low-oxygen ecosystems: the transport from oxic to anoxic depths attached to sinking particles in marine ecosystems [77], episodic oxygen intrusions in the OMZ that could support aerobic metabolism periodically [30], or the presence of oxygenic photosynthesis in the upper part of the OMZ, where the oxygen seems to be quickly used by the nitrifier communities [31]. To these explanations, we add the capability to perform oxygen production via NO-dismutation as suggested first by [32] and expanded in this study.

In the case of terrestrial ecosystems, oxygen depletion can occur when soil remains saturated with water for long periods. Different studies have shown that members of the nitrifiers, including AOA and AOB, are tolerant to extended periods of anoxia or can resist oxic-anoxic fluctuations retaining activity after 3–6 weeks [78, 79]. While AOB are known to switch to nitrifier denitrification upon anoxia, there was no evidence of soil AOA to be metabolically active during anoxia. The discovery of oxygen production upon oxygen depletion by the AOA *N. viennensis* (EN76), could potentially explain the capability of these nitrifiers to withstand oxic-anoxic fluctuations. While AOB occurrence in anoxic environments has been explained by nitrifier denitrification, here we add their capability of producing oxygen as an additional advantage in anoxic environments. However, whether nitrifier denitrification and oxygen production by AOB are related or happen under different conditions remains to be investigated.

If NO-dismutation by nitrifiers is common in nature, a fraction of the produced oxygen could be used to fuel ammonia oxidation by AOA and AOB and additionally to sustain aerobic members of the surrounding microbial community [80, 81] provided nitrite is sufficiently available.

An interesting point to further explore is if the oxygen production is just a survival mechanism that allows the cells to maintain a basal metabolism for a short amount of time or if they can sustain their energy requirements long term via NO-dismutation.

## Conclusions

In conclusion, dark oxygen production is a common trait among cultured representatives of AOA of marine and soil origin and also occurs in AOB. The coupling of oxygen production to NO accumulation and the conversion of nitrite to N_2_O, and in some cases subsequently reduction to N_2_, point towards NO-dismutation as the pathway, with some physiological differences among the AOA strains. One of the most interesting differences was the lack of N_2_O reduction by *N. adriaticus* NF5 and *N. viennensis* EN76*,* with N_2_O as final product, different from the rest of AOA strains tested (*N. piranensis* D3C, *N. adriaticus* CCS1, and *N. maritimus* SCM1), which produced N_2_. This difference adds complexity to our understanding about the role of the AOA as potential sources or sinks of the greenhouse gas N_2_O in oxygen-depleted ecosystems.

Furthermore, we presented evidence of oxygen accumulation coupled to NO accumulation in two strains of AOB upon oxygen depletion, with higher oxygen accumulation rates in comparison to AOA. It remains to be investigated whether dark oxygen production by AOA and AOB occurs in the environment and if the oxygen produced can be utilized by other community members in nature.

## Supplementary Material

202606_sup_Hernandez-Magana_AOA_NOdism_ISMEC_ycag171

## Data Availability

All data generated or analysed during this study are included in this published article and its supplementary information files.
