# Supplementary material for: Oxygen production via nitric oxide dismutation in diverse ammonia oxidizers
Source: ISME Commun. 2026 Jun 22;6(1):ycag171. doi: 10.1093/ismeco/ycag171 (PMC13464753; doi:10.1093/ismeco/ycag171)
Supplement: Supplementary file 1 [file 202606_sup_hernandez-magana_aoa_nodism_ismec_ycag171.docx]

# Supplementary material

# Oxygen production via nitric oxide dismutation in diverse ammonia oxidizers

Elisa Hernández-Magaña^1^ ^†^, Muzi Li^2^, Alyson E. Santoro ^2^, Donald E. Canfield^1^, Beate Kraft ^1†^.

^1^Nordcee. Department of Biology, Faculty of Sciences. University of Southern Denmark.

^2^Ecology, Evolution and Marine Biology. University of California Santa Barbara.

^†^Corresponding authors: Beate Kraft, Nordcee. Department of Biology, Faculty of Sciences. University of Southern Denmark. Campusvej 55, DK-5230 Odense M

Email: [bkraft@biology.sdu.dk](mailto:bkraft@biology.sdu.dk)

Elisa Hernández-Magaña, Nordcee. Department of Biology, Faculty of Sciences. University of Southern Denmark. Campusvej 55, DK-5230 Odense M

Email: [elisa@biology.sdu.dk](mailto:elisa@biology.sdu.dk)

**Supplementary Materials and Methods**

*Culture growth*

Oxygen production in the dark coupled to nitrous oxide and dinitrogen production was tested under oxygen-depletion in incubations of four marine AOA strains from the clade *Nitrosopumilales* (Group I.1a): *Nitrosopumilus adriaticus* NF5, *Nitrosopumilus piranensis* D3C, isolated from the Adriatic Sea [1,2] and *Nitrosopumilus adriaticus* CCS1, a novel strain isolated from the water column of the California Current system in the North Pacific Ocean [3]. The marine strain *N. maritimus* SCM1 [4], previously tested in [5], was also included. The soil AOA strain *Nitrososphaera viennensis* EN76 [6] from the clade *Nitrososphaerales* (Group I.1b) was tested as well*.* The AOB strains tested were *Nitrosomonas europaea* Nm50 and *Nitrosospira multiformis* Nl13 both isolated from soils*.*

The strain *N. maritimus* SCM1 (NCIMB 15022) was provided by Martin Könneke. The strain *N. piranensis* D3C (JCM 32271, DSM 106147, NCIMB 15115) and *N. viennensis* EN76 (JCM 19564, DSM 26422) were obtained from the Japan Collection of Microorganisms (JCM), the strains *N. adriaticus* NF5 (JCM 32270, NCIMB 15114) was obtained from Barbara Bayer and *N. adriaticus* CCS1 was isolated in the Laboratory of Alyson Santoro, while the strains *N. europaea* (DSM 28437) and *N. multiformis* (DSM 101674) were obtained from the DSMZ-German Collection of Microorganisms and Cell Cultures GmbH.

All the cultures were grown in axenic batch cultures in the dark and without stirring. Growth was monitored by ammonium consumption, nitrite production, and cell counts. Ammonium was measured with colorimetric salicylate-hypochlorite assay based on [7], nitrite was measured with the colorimetric detection with the Griess assay. Cell numbers were determined by fluorescence microscopy with DAPI staining.

*Oxygen-depleted incubations.*

Batch cultures that had oxidized at least 95% of the initial NH_4_^+^ (1mM) were used for incubations under oxygen depletion. Prior to the incubations, all cultures were grown aerobically. Each batch culture was sparged with argon gas (99.999% purity) for 45 min to reduce the oxygen concentration in the culture. The culture was aseptically transferred into custom-made 330 mL glass bottles [8] through a glass tube connection using the overpressure generated in the Ar-sparged culture bottle. The incubation bottles were filled completely without headspace and closed with glass stoppers. At least triplicates per treatment were used. The design of the incubation bottles makes oxygen intrusion negligible and includes a long capillary for pressure compensation [8].

Bottles were constantly stirred with glass-coated stirring bars (VWR, UK) at 300 rpm submerged in a water bath at the respective growth temperature of each strain for 72 h (Table 1 in main text). Bottles, stirring bars and tube connections were autoclaved before use. Oxygen was monitored constantly during the incubation with trace fluorescence oxygen sensors (optodes) with a lower detection limit of 0.5 nmol·l^-1^ [9] that were glued into the incubation bottles prior to utilization. NO concentrations were continuously monitored with NO microsensors (Unisense, Denmark), inserted in the sensor ports of the incubation bottles. NO-microsensors were sterilized with 70% ethanol and rinsed with autoclaved ASTM1a water before insertion into the incubation bottles. NO was previously reported to cause a small (up to 17%) and predictable interference with the optodes [5]. Therefore, all oxygen concentration measurements were corrected for NO interference following [30]. Other potential intermediates in ammonia oxidation or nitrite conversion were reported to not interfere with oxygen measurements by the optodes [5].

## Supplementary figures.

**Figure S1.** Response of oxygen producing incubations of *N. maritimus* SCM1 to different sizes of oxygen pulses. Two replicates (A, B and C, D) are shown. A and C show the first 6h of the incubations, while B and D give an overview of the whole incubation time. Arrows indicate oxygen pulses.

ALT TEXT: Graphs showing the dissolved oxygen concentration over time. Upon oxygen consumption, oxygen concentration increases, when oxygen pulses are introduced in the incubation oxygen decreases and then accumulates again. The response in oxygen production is more pronounced when oxygen pulses are higher.

**Figure S2.** Oxygen accumulation coupled to NO accumulation in oxygen-depleted incubations of AOA isolates. Oxygen concentrations are corrected for interference with NO. (A) *Nitrosopumilus maritimus* SCM1 for which oxygen production was reported before by [5]; incubations with the same strain are shown here for comparison. (B) *Nitrosopumilus piranensis* D3C. (C) *Nitrosopumilus adriaticus* NF5. (D) *Nitrosopumilus adriaticus* CCS1. Oxygen and NO profile show one out of three reproducible replicates (at least n=3 per incubation), the other replicates are shown in Figure 1. Pronounced increases in the oxygen concentration are due to oxygen intrusion by sample collection or addition of oxygenated water (see main text). Incubation of *Nitrosopumilus piranensis* D3C (B) did not have NO measurement of that specific replicate.

ALT TEXT: Graphs showing the dissolved oxygen and NO concentration over time in incubations with different ammonia-oxidizing archaea (replicates of marine strains of Figure 1 of the main text showing the same patterns: all strains accumulated oxygen upon depletion).

**Figure S3.** (A) Oxygen accumulation coupled to NO accumulation in oxygen-depleted incubations of AOA from soil *Nitrosophaera viennensis* EN76. Oxygen concentrations are corrected for interference with NO. One out of three reproducible replicates is shown, the other replicates are shown in Figure 1. Pronounced increases in the oxygen concentration are due to oxygen intrusion associated to sample collection. (B) No oxygen consumption or production was observed in killed control with medium and cells of *N. viennensis* killed with mercuric chloride. Incubated in parallel to replicates of B1-B3 presented in Figure 1 and Figure S3 A.

ALT TEXT: (A) Graph showing the dissolved oxygen and NO concentration over time in incubations with the soil AOA *N. viennensis* EN76 (replicate of experiment presented in Figure 1 of the main text showing the same pattern of oxygen production upon depletion). (B) In the controls with dead cells oxygen concentration does not change overtime, temperature is also showed as example of how the temperature during incubations is stable.

**Figure S4.** Oxygen accumulation in oxygen-depleted incubations of different AOB isolates. (A) *Nitrosomonas europaea,* (B) *Nitrosospira multiformis*. One reproducible replicate of at least n=3 per incubation is shown (Other replicates are shown in main figure 2). Pronounced increases in the oxygen concentration of (A) are due to oxygen intrusion associated to oxygenated water injection in the incubation.

ALT TEXT: Graphs showing the dissolved oxygen concentration over time in incubations with two ammonia oxidizing bacteria species (replicates of marine strains of Figure 2 of the main text showing the same patterns: all strains accumulated oxygen upon depletion).

**Figure S5.** ^29^N_2_ and ^45^N_2_O accumulation during oxygen production by different AOA strains. ^30^N_2_ and ^46^N_2_O are shown in Figure 3. ^29^N_2_ production (black triangles) and ^45^N_2_O accumulation (grey diamonds). Datapoints are an average of three replicates and error bars represent the standard deviation. Some error bars are smaller than the symbols and therefore not visible. For better interpretation of ^29^N_2_ production panel, A see main text methodology and text below. In the case of *N. viennensis*, D, no production of ^29^N_2_ was detected.

ALT TEXT: Graphs showing the ^29^N_2_ and ^45^N_2_O concentrations over time in incubations with different strains of ammonia-oxidizing archaea. Concentrations do not change overtime except for the cases of *N. piranensis* D3C which shows a slight linear increase of N_2_ and *N. viennensis* EN76 a slight increase of N2O. Explanation for these isotopic signatures is below in the supplementary note.

## Supplementary note.

Prior to the incubation of *N. piranensis* with ^15^NO_2_^-^ (Figure 3 A and Figure S5 A), 1 L of the batch culture was aerobically growth with ^14^NH_4_^+^, and when all ^14^NH_4_^+^ had been converted to ^14^NO_2_^-^, the volume of the culture was upscaled to 5 L and 1 mM of ^15^NH_4_^+^ was added. The upscaled culture was used for the incubation when more than 95% ^15^NH_4_^+^ had been converted to ^15^NO_2_^-^. Therefore, the incubation started with a pool of ca. 1 mM ^15^N-NO_2_- and 200 µM ^14^N-NO_2_^-^. *N. viennensis* batch culture started with 5% inoculum of a preculture growth with ^14^N, therefore, when starting the incubation, the culture had a pool of 1mM ^15^N-NO_2_^-^ and 50 µM of ^14^NO_2_^-^.

**Figure S6.** The phylogenetic tree of all cupredoxins in the five AOA strains. The phylogenetic tree was visualized using iTOL (v6, https://itol.embl.de/) [10]. The highlighted red clade shows the five CuA-containing proteins in the AOA strains. The highlighted blue clade shows the two CuZ-containing proteins in CCS1 and NF5. EN76 CuZ-containing protein does not have cupredoxin domains.

ALT TEXT: Phylogenetic tree with the identifiers of the proteins annotated as cupredoxins in the AOA tested.

A

B

C

**Figure S7.** Protein domains of the three CuZ-containing proteins CCS1 2932132464 (A), NF5 2630190368 (B), and EN76 2585917421 (C) visualized by online InterProScan [11] (<https://www.ebi.ac.uk/interpro/search/sequence/>).

ALT TEXT: Scheme of the proteins with CuZ domains and their position in the genomes of the compared strains CCS1, NF5 and EN76.

A

B

C

**Figure S8.** Visualization of the CuZ-containing proteins in CCS1 (A), NF5 (B), and EN76 (C) by Protter [12] (<https://wlab.ethz.ch/protter/start/>).

ALT TEXT: Scheme of the amino acid sequences in the proteins with CuZ domains of the compared strains CCS1, NF5 and EN76.

**Supplementary tables.**

**Table S1.** Hierarchical Orthologous Groups (HOG) unique to the N_2_-producing strains (CCS1, D3C and SCM1). Numbers refer to the JGI IMG gene accession number for each ortholog.

**Table S2.** Orthologs and their functions in the five orthogroups from Table S1. Annotation of CCS1 genes is shown as example, chosen for comparison with NF5 (Table S5) as they are closely related but showed physiological differences.

**Table S3.** Hierarchical Orthologous Groups (HOGs) unique to the N_2_O-producing strains (NF5 and EN76). Numbers refers to the JGI IMG gene accession number.

**Table S4.** Orthologs and their functions in the five orthogroups from Table S3. Annotation of NF5 genes is shown as example, chosen for comparison with CCS1 (Table S2) as they are closely related but showed physiological differences.

## References

1.	Bayer B, Vojvoda J, Offre P, Alves RJE, Elisabeth NH, Garcia JA *et al.* Physiological and genomic characterization of two novel marine thaumarchaeal strains indicates niche differentiation. *ISME J* 2016;**10**(5):1051–63. https://doi.org/10.1038/ismej.2015.200.

2.	Bayer B, Vojvoda J, Reinthaler T, Reyes C, Pinto M, Herndl GJ. Nitrosopumilus adriaticus sp. nov. and Nitrosopumilus piranensis sp. nov., two ammonia-oxidizing archaea from the Adriatic Sea and members of the class Nitrososphaeria. *International Journal of Systematic and Evolutionary Microbiology,* 2019;**69**(7):1892–902. https://doi.org/10.1099/ijsem.0.003360.

3.	Bayer B, McBeain K, Carlson CA, Santoro AE. Carbon content, carbon fixation yield and dissolved organic carbon release from diverse marine nitrifiers. *Limnology and Oceanography* 2022;**n/a**(n/a). https://doi.org/10.1002/lno.12252.

4.	Könneke M, Bernhard AE, Torre JR de la, Walker CB, Waterbury JB, Stahl DA. Isolation of an autotrophic ammonia-oxidizing marine archaeon. *Nature* 2005;**437**(7058):543–6. https://doi.org/10.1038/nature03911.

5.	Kraft B, Jehmlich N, Larsen M, Bristow LA, Könneke M, Thamdrup B *et al.* Oxygen and nitrogen production by an ammonia-oxidizing archaeon. *Science* 2022;**375**(6576):97–100. https://doi.org/10.1126/science.abe6733.

6.	Stieglmeier M, Klingl A, Alves RJE, Rittmann SKMR, Melcher M, Leisch N *et al.* Nitrososphaera viennensis gen. nov., sp. nov., an aerobic and mesophilic, ammonia-oxidizing archaeon from soil and a member of the archaeal phylum Thaumarchaeota. *International Journal of Systematic and Evolutionary Microbiology* 2014;**64**(Pt 8):2738. https://doi.org/10.1099/ijs.0.063172-0.

7.	Bower CE, Holm-Hansen T. A Salicylate–Hypochlorite Method for Determining Ammonia in Seawater. *Can J Fish Aquat Sci* 1980;**37**(5):794–8. https://doi.org/10.1139/f80-106.

8.	Tiano L, Garcia-Robledo E, Revsbech NP. A New Highly Sensitive Method to Assess Respiration Rates and Kinetics of Natural Planktonic Communities by Use of the Switchable Trace Oxygen Sensor and Reduced Oxygen Concentrations. *PLOS ONE* 2014;**9**(8):e105399. https://doi.org/10.1371/journal.pone.0105399.

9.	Lehner P, Larndorfer C, Garcia-Robledo E, Larsen M, Borisov SM, Revsbech NP *et al.* LUMOS - A Sensitive and Reliable Optode System for Measuring Dissolved Oxygen in the Nanomolar Range. *PLoS ONE* 2015;**10**(6):e0128125. https://doi.org/10.1371/journal.pone.0128125.

10.	Letunic I, Bork P. Interactive Tree Of Life (iTOL) v5: an online tool for phylogenetic tree display and annotation. *Nucleic Acids Research* 2021;**49**(W1):W293–6. https://doi.org/10.1093/nar/gkab301.

11.	Jones P, Binns D, Chang HY, Fraser M, Li W, McAnulla C *et al.* InterProScan 5: genome-scale protein function classification. *Bioinformatics* 2014;**30**(9):1236–40. https://doi.org/10.1093/bioinformatics/btu031.

12.	Omasits U, Ahrens CH, Müller S, Wollscheid B. Protter: interactive protein feature visualization and integration with experimental proteomic data. *Bioinformatics* 2014;**30**(6):884–6. https://doi.org/10.1093/bioinformatics/btt607.
